# Controversias en neuroinmunología: esclerosis múltiple y otras enfermedades desmielinizantes

**DOI:** 10.7705/biomedica.7882

**Published:** 2026-03-02

**Authors:** Ana C. Villegas, David Cuéllar-Giraldo, Estefanía Rojo-Bustamante, María Paula Zafra-Sierra, Lisseth Burbano, Thomas Medina, Laura Andrea Serna-Corredor, María Camila Ramírez, Daniela Sofía Rodríguez, Marcela Urbano, Aristides Duque, Jaime Toro, Saúl Reyes

**Affiliations:** 1 Programa de Neurología, Universidad El Bosque, Bogotá, D. C., Colombia Universidad El Bosque Universidad El Bosque Bogotá D. C Colombia; 2 Programa de Neurología, Fundación Santa Fe de Bogotá, Bogotá, D. C., Colombia Fundación Santa Fe de Bogotá Bogotá D. C Colombia; 3 Escuela de Medicina, Universidad de Los Andes, Bogotá, D. C., Colombia Universidad de los Andes Universidad de Los Andes Bogotá D. C Colombia; 4 Departamento de Neurología, Fundación Santa Fe de Bogotá, Bogotá, D. C., Colombia Fundación Santa Fe de Bogotá Bogotá D. C Colombia; 5 Blizard Institute, Barts and The London School of Medicine and Dentistry, Queen Mary University of London, London, United Kingdom Queen Mary University of London Queen Mary University of London London United Kingdom

**Keywords:** células madre, esclerosis múltiple, nervio óptico, posparto, recaída, Multiple sclerosis, Neuromyelitis Optica Spectrum Disorder, optic nerve, postpartum period, recurrence, stem cells, postpartum period

## Abstract

La esclerosis múltiple es la enfermedad desmielinizante más frecuente del sistema nervioso. En las últimas dos décadas, se han logrado importantes avances, como la identificación de vías inmunológicas clave, nuevos biomarcadores y el desarrollo de inmunoterapias efectivas. Sin embargo, aún persisten áreas de incertidumbre y desafíos. A nivel mundial, se ha observado un incremento notorio de los casos de esclerosis múltiple. En Colombia, por ejemplo, los estudios más recientes han reportado un cambio de la prevalencia menor de 5 por cada 100 000 habitantes a 7,5 casos por cada 100 000 habitantes. Es esencial que los neurólogos estén al tanto de los tratamientos actuales para brindar asesoramiento adecuado a estos pacientes.

Esta revisión aborda ocho controversias relevantes en esclerosis múltiple y otras enfermedades desmielinizantes, basada en la evidencia más reciente. Los temas incluyen la suspensión de tratamientos en adultos mayores, el uso de pruebas genéticas para el diagnóstico, las recomendaciones sobre planificación familiar, la prevención de recaídas posparto y el espectro de neuromielitis óptica. También se analizan la indicación de terapia con células madre, el riesgo de neoplasias malignas asociadas a la inmunoterapia, el manejo de la linfopenia y las estrategias para mitigar el efecto de rebote al suspender los tratamientos modificadores de la enfermedad. Cada controversia incluye recomendaciones para orientar la toma de decisiones en la práctica clínica diaria, adaptándose a las realidades de países como Colombia, donde son cruciales los avances en formación neurológica y acceso a tratamientos.

## Abstract (en)

Multiple sclerosis is the most prevalent demyelinating disease affecting the central nervous system. In the past two decades, significant milestones have been achieved in this domain, including the identification of critical immunological pathways, novel biomarkers, and effective immunotherapies. Nonetheless, several areas of uncertainty and challenges persist. Globally, there has been a notable increase in multiple sclerosis cases. In Colombia, recent studies indicate a rise in prevalence from fewer than 5 cases per 100 000 inhabitants to 7.5 cases per 100 000. It is imperative for neurologists to stay informed about current treatments to offer appropriate guidance to these patients. This review analyzes eight pertinent controversies surrounding multiple sclerosis, and other demyelinating diseases, based on the most current scientific evidence. Topics explored include: 1) prevention of postpartum relapses, 2) neuromyelitis optica spectrum disorder, 3) the role of genetic testing in diagnosis, 4) risks of malignancy associated with immunotherapy, 5) discontinuation of treatment in older adults, 6) management strategies for lymphopenia, 7) what is the indication for stem cell therapy in multiple sclerosis? and 8) approaches to mitigate rebound effects after discontinuing disease-modifying therapy. Each controversy is supported by a thorough analysis and practical recommendations aimed at informing decision-making in daily clinical practice and adapting to the realities of countries like ours, where advancements in neurological training and access to treatments are crucial.

La esclerosis múltiple es la enfermedad desmielinizante más común del sistema nervioso. En las últimas dos décadas, se han logrado avances significativos en el diagnóstico y el tratamiento de esta enfermedad, incluyendo la identificación de las vías inmunológicas fundamentales, los nuevos biomarcadores y el desarrollo de inmunoterapias efectivas. No obstante, aún persisten incertidumbres y desafíos.

A nivel mundial, se ha observado un notable incremento de los casos de esclerosis múltiple. En Colombia, los estudios recientes han reportado un cambio en la prevalencia, la cual pasó de menos de 5 por cada 100 000 habitantes a 7,5 casos por cada 100 000. Teniendo en cuenta este aumento, es esencial que los neurólogos estén informados sobre los tratamientos actuales para, así, ofrecer un asesoramiento adecuado a los pacientes.

En esta revisión se examinan, con base en la evidencia más reciente, las ocho controversias que se mencionan a continuación y que son relevantes en la esclerosis múltiple y en otras enfermedades desmielinizantes:

1. la prevención de las recaídas posparto,
2. el espectro de la neuromielitis óptica,
3. el uso de pruebas genéticas para el diagnóstico,
4. el riesgo de neoplasias malignas asociado con la inmunoterapia,
5. la suspensión de los tratamientos en los adultos mayores,
6. el manejo de la linfopenia,
7. la indicación de la terapia de células madre en la esclerosis múltiple, y
8. cómo evitar el efecto de rebote al suspender algunos tratamientos que modifican la enfermedad.

Cada controversia se acompaña de recomendaciones prácticas para orientar la toma de decisiones en la práctica clínica diaria y lograr adaptarse a las realidades de los países donde son cruciales los avances en la formación neurológica y el acceso a los tratamientos.

## ¿Se recomienda implementar un tratamiento específico para prevenir las recaídas en el posparto de las pacientes con esclerosis múltiple?

La esclerosis múltiple es una enfermedad que afecta predominantemente a las mujeres en edad fértil, con una relación de 3:1 respecto a los hombres. En cuanto al factor etario, el 90 % de las pacientes tiene entre 15 y 50 años ^1^. En la mayor parte de Latinoamérica, la enfermedad se presenta con particularidades geográficas, raciales y genéticas; sin embargo, Panamá y Ecuador se clasifican como regiones de bajo riesgo para la enfermedad, con menos de 5 casos por cada 100 000 habitantes ^2^^,^^3^. Colombia podría ahora considerarse una región de riesgo intermedio con 7,5 casos por cada 100 000 habitantes ^4^.

Desde 1998, con el estudio *Pregnancy in Multiple Sclerosis* (PRIMS) se ha documentado una reducción de la tasa de recaídas durante el embarazo en las pacientes de origen europeo, particularmente en el tercer trimestre; sin embargo, se ha observado un fenómeno de rebote durante los tres primeros meses después del parto, que supera la tasa basal de recaídas antes del embarazo ^5^. En consecuencia, el estudio de posibles estrategias específicas para prevenir las recaídas posparto ha cobrado relevancia. En la literatura se destacan tres enfoques principales: 1) los corticoides a grandes dosis, 2) la inmunoglobulina y 3) las hormonas sexuales.

En relación con los corticoides a grandes dosis, se han realizado estudios observacionales retrospectivos en los que se administraba 1 g mensual de metilprednisolona durante 1 a 6 meses en el periodo posparto. En un primer estudio, se reportó una tasa de recaídas posparto significativamente menor en las pacientes que habían recibido corticoides, en comparación con el grupo no tratado (0,8 ± 0,41 vs. 2 ± 0,66; p = 0,018) ^6^.

De manera similar, en otro estudio se encontró un porcentaje menor de pacientes con recaídas en el grupo tratado, en comparación con el grupo no tratado (17,9 % vs. 46,2 %; p = 0,0448) ^7^. Sin embargo, en un tercer estudio, de tipo multicéntrico y con una mayor cantidad de pacientes, no se observó un beneficio estadísticamente significativo del tratamiento, con evidencia de nivel III ^8^.

Por último, con los datos de una cohorte de 142 embarazos de 128 mujeres de Brasil, la más grande de Latinoamérica hasta el momento, se encontró una disminución significativa de la tasa de recaídas durante el posparto, en comparación con el año anterior al embarazo. Aunque en esta población se reportó el uso regular de corticosteroides e inmunoglobulinas en el posparto inmediato, la falta de consenso sobre su uso y el diseño del estudio, no permiten establecer conclusiones contundentes sobre los riesgos y los beneficios de esta práctica ^9^.

Respecto al tratamiento con inmunoglobulina endovenosa, en una revisión sistemática y un metaanálisis que incluyó 380 pacientes, no se logró establecer un efecto terapéutico claro, y se indicó que la variabilidad de los esquemas terapéuticos y de la naturaleza de los estudios incluidos, había sido una limitación ^10^. Sin embargo, en un estudio observacional retrospectivo más reciente, se encontró que las pacientes no tratadas tenían un riesgo 4,6 veces mayor de recaída posparto en comparación con aquellas que habían recibido tratamiento con inmunoglobulina endovenosa [OR = 4,6; IC_95%_: 1,69 - 12,78); p = 0,033] ^11^.

En cuanto a las hormonas sexuales, en un ensayo clínico aleatorizado, multicéntrico y doble ciego, se reportó que no se habían encontrado beneficios en la tasa de recaídas al usar estrógenos más progestágenos, en comparación con el grupo que había recibido placebo [OR = 0,90; IC_95%_: (0,58 a 1,39) *vs.* OR = 0,97, IC_95%_: (0,63 a 1,50); p = 0,79] ^12^.

Por otro lado, la lactancia exclusiva se ha asociado con una reducción del riesgo de recaídas posparto. En un metaanálisis se indicó una disminución del 43 % en la tasa de recaídas en 2974 mujeres que optaron por brindarle lactancia exclusiva al bebé durante los primeros seis meses de vida ^13^. En otro metaanálisis que incluyó 2739 embarazos, se reportó que la lactancia exclusiva y el uso previo de ciertas terapias modificadoras de la enfermedad se asociaba con una reducción significativa del riesgo de recaídas en el periodo posparto ^14^.

El interferón beta y el acetato de glatiramer han demostrado reducir el riesgo de recaídas en el posparto, especialmente en mujeres que no amamantan, aunque su inicio temprano no parece modificar significativamente el riesgo en los primeros seis meses ^15^. Por su parte, el natalizumab, continuado hasta las semanas 32 a 34 de gestación y reanudado tempranamente tras el parto, se asoció con una disminución importante de la actividad de la enfermedad ^16^. Si bien la evidencia sobre el alemtuzumab y el anti-CD20 es limitada, algunos estudios sugieren que podrían conferir un efecto protector en el periodo posparto ^17^.

La suspensión de los tratamientos modificadores de la enfermedad incrementa el riesgo de recaídas ^18^, las cuales pueden llegar a ser graves, como en el caso del fingolimod ^19^^,^^20^, razón por lo cual resulta fundamental su reintroducción oportuna tras el parto. Esta decisión debe individualizarse por medio de una valoración del riesgo de reactivación de la enfermedad, la seguridad durante la lactancia y las preferencias de la paciente, considerando que hasta el 25 % de las mujeres con esclerosis múltiple puede presentar recaídas en los primeros tres meses después del parto ^21^^-^^23^.

En conclusión, la evidencia actual no es suficiente para recomendar el uso preventivo de corticoides, inmunoglobulina intravenosa u hormonas sexuales para disminuir las recaídas en el posparto en las pacientes con esclerosis múltiple. La mayoría de los estudios disponibles son observacionales e incluyen pocas poblaciones hispanas y latinoamericanas.

La suspensión de las terapias modificadoras de la enfermedad aumenta el riesgo de recaídas ^21^^-^^23^, por lo que deben reiniciarse oportunamente después del parto, individualizando cada caso de acuerdo con el efecto de la lactancia y las preferencias de la paciente. La lactancia materna exclusiva se ha asociado con una reducción del riesgo de recaídas posparto.

Es crucial continuar investigando en esta área, ya que el desarrollo de estrategias preventivas podría mitigar la carga de la enfermedad, reducir los costos y optimizar los recursos de los sistemas de salud. Además, al tratarse de una población vulnerable que enfrenta barreras adicionales en los países de los países de medianos o bajo ingresos, la implementación de estrategias de prevención de las recaídas resultaría especialmente beneficiosa.

## ¿Se pueden mitigar los riesgos relacionados con el embarazo en pacientes con esclerosis múltiple y el espectro de neuromielitis óptica?

En Colombia, se llevó a cabo un estudio de corte transversal, que incluyó a 141 mujeres, en el que el 36,9 % reportó el deseo de tener hijos. En esta población se identificaron diferentes necesidades insatisfechas en el abordaje de la planeación familiar. En general, para la población colombiana se ha estimado que hasta el 67 % de los embarazos no son planeados. Por esta razón, es importante que los neurólogos estén familiarizados con las estrategias para mitigar los riesgos relacionados con el embarazo de las pacientes con esclerosis múltiple o con el espectro de neuromielitis óptica ^24^^,^^25^.

En primer lugar, se sugiere planificar con suficiente tiempo de antelación y después, al menos, de 12 meses de inactividad de la enfermedad ^24^^,^^25^. Durante las consultas con el neurólogo, es importante discutir regularmente el deseo de maternidad y la anticoncepción. Antes del embarazo, es crucial consolidar el manejo farmacológico y actualizar el esquema de vacunación, siguiendo las pautas para la población general. Las vacunas vivas atenuadas están contraindicadas durante el embarazo, pero pueden administrarse después del parto y antes de iniciar cualquier tratamiento que modifique la enfermedad ^26^^,^^27^.

En cuanto a los riesgos obstétricos, en las mujeres embarazadas que padecen esclerosis múltiple, se observa un aumento significativo de las infecciones urinarias y la exacerbación de algunos síntomas residuales, como la espasticidad y la fatiga. Para las mujeres con trastorno del espectro de neuromielitis óptica ^25^, dada su asociación con otras enfermedades autoinmunitarias, del 20 al 30 % ^25^, se ha reportado un mayor riesgo de abortos, preeclampsia y retraso del crecimiento intrauterino, así como recaídas durante el embarazo y el posparto ^28^.

En un estudio se reportó una tasa de abortos del 42,9 % en los embarazos posteriores al diagnóstico, en comparación con el 7,04 % en los embarazos anteriores al inicio de la enfermedad ^29^. Además, se observó una mayor incidencia de complicaciones obstétricas, como preeclampsia y partos prematuros ^28^.

En Tailandia, otro estudio retrospectivo reveló que el 76,9 % de las recaídas ocurrieron en el posparto, con un aumento significativo de la tasa anual de recaídas durante los 12 meses después del parto. Además, el 66,7 % de los embarazos resultaron en recaídas posparto, de los cuales la mayoría (88,7 %) ocurrió en los primeros tres meses después del parto ^30^.

Asimismo, es necesario distinguir entre los síntomas que sugieren una recaída de la enfermedad y aquellas condiciones neurológicas relacionadas con la gestación, como la parálisis de Bell, las neuropatías por atrapamiento, la meralgia parestésica y el síndrome del túnel del carpo ^5^^,^^31^.

Durante el embarazo y en el periodo posparto, se recomienda establecer un manejo multidisciplinario que incluya consultas trimestrales con neurología y obstetricia, con seguimiento ecográfico y adaptado a las necesidades individuales de cada paciente.

Los objetivos específicos del seguimiento neurológico durante el embarazo y en el posparto incluyen:

- identificar el inicio o el empeoramiento de cualquier síntoma neurológico,
- determinar la necesidad de un control imagenológico,
- decidir el tratamiento para una recaída y, cuando aplique,
- organizar una atención adaptada al grado de la discapacidad.

No se recomienda practicar resonancias magnéticas de control de manera rutinaria; sin embargo, en caso de ser necesarias, se pueden llevar a cabo, incluso con la administración de gadolinio, independientemente del trimestre ^2^^,^^24^^,^^25^.

Las decisiones sobre el parto, la analgesia y la anestesia no difieren de las prácticas estándar del manejo obstétrico ^32^.

Algunos de los tratamientos que modifican la enfermedad se asocian con un mayor riesgo de teratogenicidad. Aquellos que se consideran más seguros durante el embarazo incluyen: interferones, acetato de glatiramer, natalizumab y ocrelizumab ^16^^,^^33^. Para los fármacos que aumentan el riesgo de teratogenicidad, como es el caso de la teriflunomida, el alemtuzumab y la cladribina, se deben observar las recomendaciones sobre los tiempos de espera antes de la concepción. Se recomienda un periodo de espera de 48 meses para la teriflunomida, de 6 meses para el alemtuzumab y de 4 a 6 meses para la cladribina, dado su potencial genotóxico y el riesgo de efectos adversos en el feto ^17^^,^^34^.

La lactancia materna no está contraindicada y, de hecho, podría tener un efecto protector contra las recaídas posparto. Los estudios han sugerido que la lactancia, especialmente cuando es exclusiva, puede asociarse con una menor tasa de recaídas en el periodo posparto ^13^.

Si los tratamientos modificadores de la enfermedad se suspendieron durante el embarazo, se deben reanudar prontamente en el periodo posparto para evitar recaídas, ya que el 25 % de las mujeres experimenta recaídas en los primeros tres meses después del parto ^21^^-^^23^. No obstante, la reanudación temprana de dichos tratamientos debe ser cuidadosamente considerada en función del riesgo de reactivación de la enfermedad y de las preferencias de la paciente, ya que el paso de ciertos fármacos a la leche materna y la exposición subsecuente del lactante pueden representar riesgos ^35^. La cladribina, el alemtuzumab, la mitoxantrona y el micofenolato pueden pasar a la leche materna ^31^, aunque la dosis relativa que alcanzan en el lactante es baja. A pesar de esto, antes de reanudar la lactancia, se sugiere esperar, al menos, una semana tras la última dosis de la cladribina ^36^, de 4 a 6 meses para el alemtuzumab y la mitoxantrona, y de 6 semanas para el micofenolato ^24^^,^^25^.

Respecto al manejo de las recaídas durante el embarazo, se recomienda un ciclo corto de metilprednisolona a dosis altas ^8^. Para evitar complicaciones durante la organogénesis, se recomienda, si es posible, comenzar el tratamiento con los corticosteroides después de la semana 10 del embarazo. Si se trata de una recaída en el posparto, se sugiere esperar de 2 a 4 horas después de la administración de los esteroides antes de reiniciar la lactancia, con el fin de limitar la exposición del infante ^22^. Si hay una recaída importante que no responde a los corticosteroides, es posible considerar la plasmaféresis. Por último, en las pacientes con esclerosis múltiple, la reproducción asistida debe ofrecerse con precaución y en coordinación entre los especialistas correspondientes ^31^.

A pesar de la abundante literatura global sobre la esclerosis múltiple durante el embarazo, la información sobre el manejo de la salud reproductiva en las pacientes del espectro de trastorno del espectro de neuromielitis óptica, es escasa y se basa en informes de casos, pequeños estudios retrospectivos, opiniones de expertos y evidencia clínica no sistemática, lo cual resalta la necesidad de más estudios en este campo ^24^^,^^25^.

En conclusión, el embarazo no está contraindicado en las pacientes con esclerosis múltiple o trastorno del espectro de neuromielitis óptica ^24^^,^^25^; de hecho, la mayoría de las pacientes logra llevar a término embarazos saludables. Sin embargo, es fundamental que reciban una atención especializada que incluya una planificación cuidadosa. También, lo es un enfoque multidisciplinario para manejar los riesgos obstétricos y neurológicos, así como para administrar los tratamientos de manera segura. Además, la vigilancia constante durante el embarazo y el periodo posparto contribuye a optimizar los resultados, tanto para la madre como para el neonato.

## ¿Es útil solicitar pruebas genéticas a los pacientes con esclerosis múltiple?

La esclerosis múltiple es una enfermedad de herencia compleja ^37^^,^^38^. Cerca del 15 % de los pacientes reporta tener, al menos, un familiar con la enfermedad y se estima que los factores de riesgo genético pueden explicar entre el 30 y el 50 % de la propensión a la enfermedad ^37^^,^^39^. Estos factores determinantes genéticos interactúan con otros, como la deficiencia de vitamina D, el tabaquismo, la obesidad, la infección por el virus de Epstein-Barr y las alteraciones en el microbioma, que operan de manera sinérgica para favorecer el desarrollo de la enfermedad ^37^^,^^40^.

En las últimas décadas, los estudios genéticos como los de análisis de ligamiento familiar y los estudios de asociación del genoma completo *(Genome-Wide Association Studies,* GWAS), han permitido identificar variantes genéticas protectoras y de riesgo para el desarrollo de la enfermedad, con lo cual se ha mejorado la comprensión de sus mecanismos biológicos ^37^^,^^41^. Se han identificado más de 200 *loci* de susceptibilidad, incluidas 32 variantes en el *locus* del complejo mayor de histocompatibilidad (CMH), que pueden explicar hasta la mitad de la heredabilidad de la esclerosis múltiple ^39^^,^^41^.

Las asociaciones genéticas más fuertes se relacionan con los polimorfismos de los genes del antígeno leucocitario humano *(Human Leukocyte Antigens,* HLA), especialmente la variante HLA-DRB1*15:01, que triplica el riesgo de desarrollar la enfermedad ^40^^,^^42^, al afectar los mecanismos de reparación del sistema nervioso central (42). También, se ha encontrado que variantes como la DYSF-ZNF638 (rs10191329) están asociadas con una progresión más rápida de la enfermedad.

El riesgo de desarrollar esclerosis múltiple varía según la relación familiar. En gemelos monocigóticos, la concordancia puede alcanzar entre el 25 y el 30 %; en gemelos dicigóticos es del 6 % y entre hermanos del 2 al 4 % ^38^^,^^39^. Sin embargo, estos datos indican que, aunque los factores genéticos son importantes, no son determinantes por sí solos. Curiosamente, algunos factores genéticos asociados a la esclerosis múltiple, como ciertas variantes del HLA introducidas por poblaciones antiguas (por ejemplo, los pastores yamnaya de hace 5000 años), ofrecieron ventajas frente a infecciones en su época, pero en el contexto moderno aumentan el riesgo de enfermedades autoinmunitarias como la esclerosis múltiple ^43^.

La diversidad genética de Latinoamérica varía según la ascendencia indígena y europea, influenciada por las diferentes poblaciones colonizadoras de cada país. Esto hace imprescindible conocer los factores genéticos de riesgo y de protección de nuestras poblaciones ^44^^,^^45^.

En un estudio mexicano se encontró que los pacientes mestizos con diagnóstico de esclerosis múltiple presentan proporciones genéticas distintas a las de personas sanas, lo que sugiriere que existe un mayor componente europeo en la susceptibilidad a la enfermedad ^4^. A su vez, en Brasil, se han identificado haplotipos relacionados con la esclerosis múltiple: el HLA-DRB1*15:01 y el polimorfismo CIITA de +1614G/C se asocian con una mayor frecuencia de la enfermedad ^46^. En Argentina, se replicó el hallazgo del HLA-DRB1*15:01, mientras que HLA-DRB114 mostró ser un efecto protector, aunque no fue estadísticamente significativo (p = 0,07) ^47^. Posteriormente, en un estudio de nuestro grupo de investigación de una población colombiana, se validó esta asociación protectora y el resultado fue estadísticamente significativo ^40^.

Hoy en día, el uso de pruebas genéticas en la esclerosis múltiple se limita al ámbito de la investigación. Aunque estas pruebas han aportado información valiosa sobre la susceptibilidad y los mecanismos biológicos de la enfermedad, no son herramientas diagnósticas en la práctica clínica diaria ^48^^,^^49^.

Las variantes conocidas no permiten confirmar el diagnóstico ni predecir la progresión de manera concluyente en cada paciente. Por esta razón, el diagnóstico sigue basándose en los criterios de McDonald, que integran los hallazgos clínicos, los estudios de neuroimágenes y el análisis del líquido cefalorraquídeo ^50^. No obstante, los avances en genética y en tecnología podrían transformar el manejo de la esclerosis múltiple en el futuro.

Es posible que las pruebas genéticas faciliten un diagnóstico temprano en los pacientes con alto riesgo o que permitan desarrollar tratamientos personalizados, optimizándolos según el perfil genético del paciente. Además, la inteligencia artificial y los sistemas de predicción basados en grandes bases de datos genéticos, podrían ayudar a identificar las variantes relacionadas con la progresión de la enfermedad y a crear intervenciones más efectivas ^48^^,^^49^.

En conclusión, no hay evidencia que respalde la solicitud de practicar pruebas genéticas de manera rutinaria en los pacientes con diagnóstico de esclerosis múltiple. No obstante, su papel en la investigación sigue siendo crucial y debe estudiarse en cada población, especialmente en las zonas donde la ocurrencia y las características de la enfermedad son muy heterogéneas, como en Latinoamérica ^44^.

## ¿Existe riesgo en los pacientes con esclerosis múltiple de desarrollar cáncer con las terapias modificadoras de la enfermedad?

El impacto de este tipo de tratamientos sobre el riesgo de cáncer en los pacientes con esclerosis múltiple es un área de investigación continua. Se ha propuesto que la inflamación crónica en la esclerosis múltiple y la administración de estos tratamientos podrían aumentar el riesgo de neoplasias ^51^. Se proyecta que para el año 2040 habrá un incremento del 60 % de los casos de cáncer en Colombia y del 70 % del número de muertes relacionadas con esta condición. Por esta razón, es importante conocer los posibles riesgos respecto al desarrollo de cáncer asociados con este tipo de terapias. Lo anterior tiene un impacto directo en la práctica clínica, en cuanto a la estimación de riesgos y tamizajes en aquellos pacientes que iniciarían o que ya se encuentran recibiendo terapias modificadoras de la enfermedad.

En un estudio de 5146 pacientes con esclerosis múltiple en Canadá, se evaluó el riesgo de cáncer asociado con el tratamiento con interferón beta (IFNß) durante un periodo de observación de 12 años. Aunque los resultados no mostraron un aumento significativo del riesgo general de cáncer, se observó una tendencia no significativa hacia un mayor riesgo de cáncer de mama en los pacientes tratados ^52^. En otro estudio multicéntrico en Europa, no se encontró un aumento del riesgo, mientras que, en un análisis de farmacovigilancia de la Organización Mundial de la Salud (OMS) (2000-2019), se reportó una asociación significativa entre el uso prolongado de interferón beta y el cáncer (OR = 1,39; IC_95%_: 1,30-1,49), especialmente para tumores sólidos ^53^. En general, se considera que el riesgo de cáncer con la administración de interferón beta es bajo.

El acetato de glatiramer presenta un buen perfil de seguridad, sin casos de cáncer reportados en los estudios que sirvieron como pilares de investigación para su desarrollo clínico ^53^. En un estudio israelí posterior a su comercialización, se indicó una tendencia no significativa hacia un incremento del riesgo de cáncer de mama. Si bien se han documentado condiciones cutáneas y malignas, como melanoma y linfoma, con los estudios de farmacovigilancia se no ha encontrado un aumento significativo de su incidencia ^54^.

En cuanto a la teriflunomida, en los estudios pilares relacionados con este medicamento, se reportó un caso de cáncer de cuello uterino y otro de leiomiosarcoma entre 876 pacientes, sin un aumento significativo del riesgo en los estudios posteriores a su comercialización ^55^^,^^56^.

Respecto al dimetilfumarato, en el estudio DEFINE se reportaron cuatro casos de neoplasias ^57^, mientras que, en un análisis de farmacovigilancia de la OMS, se encontró un riesgo elevado de cáncer, particularmente de mama y de piel ^53^; sin embargo, otros estudios no respaldan esta tendencia 54.

El fingolimod se ha asociado principalmente con el cáncer de piel, siendo el carcinoma de células basocelulares el más común, seguido por el carcinoma de células escamosas y el melanoma, según los estudios FREEDOMS II, TRANSFORMS e INFORMS ^58^^,^^59^. También, se reportaron otros tumores sólidos, que representaron el 3,28 % de los pacientes tratados; sin embargo, en un estudio de farmacovigilancia de la OMS, no se consideró que el riesgo fuera significativamente mayor al esperado en la población general ^58^^,^^59^.

Por su parte, la cladribina fue rechazada por la *Food and Drug Administration* (FDA) en el 2010 debido a preocupaciones sobre el riesgo de cáncer; no obstante, el estudio CLARITY reportó únicamente 10 casos de neoplasias. En un metaanálisis se concluyó que no producía un riesgo aumentado en comparación con otros tratamientos ^34^^,^^60^, hallazgo similar al observado en los pacientes con leucemia tratados con cladribina, en quienes no se encontró un incremento del riesgo de neoplasias secundarias ^34^^,^^60^.

En el estudio AFFIRM ^61^, que incluyó 942 pacientes, se reportaron solo 5 casos de cáncer en el grupo tratado con natalizumab. En un estudio de farmacovigilancia, se reportó una probabilidad aumentada de cáncer (OR = 1,74; IC_95%_: 1,63-1,87) en los pacientes tratados con natalizumab, aunque en otros estudios no se encontró un riesgo elevado ^54^^,^^60^. Además, se está investigando si el natalizumab podría tener un potencial terapéutico para algunos tipos de cáncer o si, por el contrario, podría acelerar la progresión del linfoma en el sistema nervioso central ^51^^,^^60^.

Generalmente, el alemtuzumab se considera seguro respecto al riesgo de neoplasias malignas. En los estudios CARE-MS-I ^62^ y CARE-MS-II ^63^, que incluyeron 810 pacientes, se reportaron 10 casos de neoplasias, y el cáncer papilar de tiroides fue el más común. Aunque no se ha evidenciado un aumento significativo del riesgo de cáncer con este medicamento, se han documentado casos de progresión acelerada de un cáncer subyacente ^55^.

En los estudios OPERA I y II ^64^ y ORATORIO ^65^ que incluyeron 1311 pacientes tratados con ocrelizumab, se reportaron 15 neoplasias, y el cáncer de mama fue el más frecuente; sin embargo, en un estudio de seguridad a siete años, no se halló un riesgo aumentado de neoplasias malignas.

En el estudio ALITHIOS, con el que se evaluó la seguridad del ofatumumab en la esclerosis múltiple recurrente hasta por 3,5 años, se observó un riesgo bajo de cáncer, con una incidencia del 0,6 %, principalmente, de carcinomas basocelulares y algunos casos de cáncer de mama. Estos datos indican que la administración prolongada del ofatumumab no incrementa la incidencia de neoplasias malignas ^66^.

A la luz de lo expuesto anteriormente, en un estudio del 2021 se evaluaron los datos de la FDA sobre todos los medicamentos mencionados desde el 2004 hasta el 2020, y se encontró que ninguno producía un riesgo desproporcionado de cáncer en los pacientes con esclerosis múltiple ^51^. En general, se considera que las terapias modificadoras de la enfermedad no incrementan de forma significativa el riesgo de cáncer en los pacientes con esclerosis múltiple. Sin embargo, se debe hacer un tamizaje adecuado y controlar otros factores de riesgo para neoplasias. Además, se sugiere considerar un seguimiento más estricto para los pacientes que reciben dimetilfumarato, fingolimod, cladribina y o natalizumab ^54^.

## ¿Se debería suspender la terapia modificadora de la enfermedad en los pacientes adultos mayores con esclerosis múltiple?

Los pacientes con esclerosis múltiple experimentan una disminución de la actividad inflamatoria de la enfermedad con la edad, debido a un proceso natural de inmunosenescencia ^67^. Esto es relevante, ya que la mayoría de los tratamientos que modifican la enfermedad se utilizan para controlar dicha actividad inflamatoria y han sido ampliamente estudiados en los pacientes menores de 55 años ^68^.

La suspensión de este tipo de tratamiento en los pacientes mayores de 55 años sin actividad de la enfermedad es un tema de constante debate. Usualmente, se discuten sus altos costos, el perfil de seguridad en este grupo etario, la creciente población anciana y la necesidad de optimizar los recursos de salud ^67^. Existen argumentos que señalan la falta de evidencia clara sobre los desenlaces de la enfermedad al suspenderlos, especialmente, en el caso de tratamientos que se asocian con un fenómeno de rebote de la actividad inflamatoria. Además, la mayoría de los estudios en que se ha evaluado la suspensión de estas terapias modificadoras presentan limitaciones metodológicas, lo que disminuye la posibilidad de obtener conclusiones definitivas ^67^.

Recientemente, se publicó el DISCOMS, primer estudio clínico aleatorizado para evaluar la suspensión de las terapias modificadoras de la enfermedad en los pacientes con esclerosis múltiple. Los pacientes incluidos eran mayores de 55 años y no presentaban evidencia de actividad radiológica ni clínica de la enfermedad en los últimos 3 y 5 años, respectivamente. Durante un periodo de seguimiento de 24 meses, los resultados del estudio no lograron refutar la hipótesis nula de que la suspensión de estos tratamientos era inferior a su continuación. Además, no se identificaron factores específicos asociados con un mayor riesgo de recaídas tras su suspensión ^64^.

En línea con lo anterior, en una revisión sistemática y un metaanálisis de estudios de la vida real, se encontró que la tasa de recaídas y la progresión de la enfermedad al suspender las terapias modificadoras eran muy bajas (menos del 1 %) en aquellos pacientes mayores de 60 años con exposición al tratamiento por 10 o más años o estabilidad de la enfermedad de 8 años o más años ^69^.

Se debe individualizar la decisión de suspender los tratamientos modificadores de la enfermedad en los pacientes mayores de 55 años sin actividad de la enfermedad. Esta conducta podría ser segura en aquellos pacientes con exposición prolongada al tratamiento, de 5 a 10 años, y con enfermedad inactiva durante más de 5 años. Esto es relevante en los contextos locales y en los países de ingresos de medianos a bajos, donde el acceso a los tratamientos puede ser limitado y los costos son una preocupación constante ^69^. Sin embargo, se deben considerar las características demográficas y epidemiológicas de la población local, así como la variabilidad en el acceso y la calidad de la atención médica.

Se requieren más estudios para orientar adecuadamente la práctica clínica y garantizar que se tomen decisiones basadas en la evidencia, especialmente, en los pacientes que reciben terapias modificadoras de la enfermedad muy efectivas. Mientras tanto, es crucial que la decisión sea compartida con el paciente, enfatizando la importancia de un seguimiento clínico estrecho si se suspende el medicamento.

## ¿Cómo vigilar la linfopenia en los pacientes con esclerosis múltiple que reciben terapias modificadoras de la enfermedad?

La mayoría de este tipo de tratamientos para la esclerosis múltiple, afectan el sistema inmunológico periférico, específicamente los linfocitos T y B ^70^. Estas terapias ejercen su efecto inmunomodulador al interferir con la activación, proliferación y liberación de citocinas, así como al afectar la migración de los linfocitos, lo que resulta en linfopenia periférica en algunos casos ^70^. La linfopenia puede ser un efecto deseado, y reflejar la efectividad y adhesión a algunas de ellas; sin embargo, también puede representar un efecto adverso que incrementa el riesgo de infecciones oportunistas ^71^. Por lo tanto, la valoración de los linfocitos y el criterio para suspender el tratamiento debido a la linfopenia, dependen del mecanismo de acción del medicamento y del perfil de riesgo de cada paciente ^72^.

La linfopenia puede ser un efecto adverso no deseado del tratamiento con dimetilfumarato y fingolimod. En cuanto al dimetilfumarato, aproximadamente, el 9 % de los pacientes presenta linfopenia de grado 1, el 21 % de grado 2, el 7 % de grado 3 y menos del 1 % de grado 4. Además, este trastorno es persistente en 10 a 20 % de los pacientes y es más común en pacientes mayores de 55 años, con disminución del número basal de linfocitos antes de iniciar la terapia o que hayan recibido natalizumab recientemente ^72^^,^^73^.

En los estudios pilares, se observó que el número de linfocitos disminuye hasta un 30 % en el primer año de tratamiento ^72^. Se recomienda hacer un seguimiento clinico con hemograma cada dos meses durante el primer año y, posteriormente, cada tres a seis meses ^71^. Además, se debe suspender el tratamiento si la linfopenia es inferior a 500 por μl o si se mantiene por debajo de 800 por μl durante seis meses o más.

Respecto al fingolimod, se ha reportado que la linfopenia depende de la dosis y que ocurre hasta en el 80 % de los pacientes, y es grave (grado 4) en cerca del 20 % de los casos ^71^. En general, se ha registrado una reducción del 20 al 30 % en el número de linfocitos respecto a los valores basales. Por lo tanto, se sugiere obtener un hemograma inicial, seguido de uno cada mes durante tres meses y, luego, cada tres meses durante el primer año. Posteriormente, se puede hacer un control cada tres a seis meses y se debe suspender el tratamiento en caso de presentarse linfopenia sostenida inferior a 200 por μl ^71^.

El natalizumab es un anticuerpo monoclonal que, debido a su mecanismo de acción, impide la migración de linfocitos hacia el sistema nervioso central, lo que puede generar linfocitosis periférica. Esto refleja una respuesta al medicamento y suele estabilizarse tras tres a seis meses de tratamiento ^71^^,^^72^. Por lo anterior, la linfopenia no es un efecto secundario esperado en estos pacientes; sin embargo, se recomienda practicar hemogramas basales: uno cada tres meses durante el primer año y, luego, cada tres a seis meses ^71^^,^^72^.

La inmunosupresión inducida por tratamientos como el natalizumab y el fingolimod, puede alterar la proporción de linfocitos CD4+/CD8+, lo que se ha asociado con un mayor riesgo de infecciones oportunistas ^73^, encefalitis por el virus de la varicela-zóster (VSV) o por el herpes simple (HSV) e, incluso, la leucoencefalopatía multifocal progresiva causada por el virus John Cunningham (JCV) ^74^^,^^75^. La deficiencia aislada de linfocitos CD8+ también se ha relacionado con casos de leucoencefalopatía multifocal progresiva ^76^, destacándose la importancia de una vigilancia inmunológica adecuada de los pacientes que estén recibiendo estas terapias.

Otros anticuerpos monoclonales, como el ocrelizumab, tampoco suelen tener un efecto significativo sobre el número de linfocitos periféricos ^70^. La linfopenia inducida por el ocrelizumab puede presentarse en el 20 % de los pacientes y suele ser de grado 1 o grado 2; cuando se presenta linfopenia grave, se deben buscar otras causas ^71^^,^^72^. De manera similar a lo que se hace con otros tratamientos que modifican la enfermedad, se recomienda solicitar un hemograma basal y, posteriormente, uno cada tres meses ^71^.

El tratamiento con anticuerpos anti-CD20, como el rituximab y el ocrelizumab, causa depleción de células B y pre-B por 6 a 12 meses, y su uso crónico puede inducir citopenias, hipogammaglobulinemia y predisposición a linfomas asociados con el virus de Epstein-Barr, lo cual aumenta el riesgo de infecciones por agentes patógenos oportunistas, toxoplasma y virus JCV ^77^^-^^79^. En el caso del eculizumab en pacientes con trastorno del espectro de neuromielitis óptica, se ha informado que este tratamiento incrementa el riesgo de infecciones por agentes patógenos encapsulados, especialmente *Neisseria meningitidis,* mientras que el alemtuzumab se asocia con un mayor riesgo de enfermedades autoinmunitarias e infecciones oportunistas, como aquellas por el HSV o las bacterias *Listeria* sp. o *Nocardia* sp.

La determinación regular de la concentración de las inmunoglobulinas es esencial. En casos de hipogammaglobulinemia significativa con infecciones graves y recurrentes, se recomienda evaluar los valores de IgG, IgA e IgM cada seis meses ^80^. Asimismo, se aconseja considerar la terapia de reemplazo con inmunoglobulina intravenosa en aquellos pacientes con disminución de la IgG (<5 g/dl), y con antecedentes de infecciones graves y recurrentes, para reducir el riesgo infeccioso y mejorar el pronóstico clínico ^76^^,^^80^^,^^81^.

En el caso de los tratamienetos de reconstitución inmunológica, la linfopenia puede ser un efecto deseado e indicar una respuesta terapéutica adecuada. La gran mayoría de los pacientes tratados con alemtuzumab van a presentar una linfopenia de grado 3 o 4 en el primer mes y, en menor medida, pueden desarrollar otras citopenias como neutropenia, que es leve en el 16 % y grave en menos del 1 % de los casos ^71^^,^^72^. Por lo anterior, se recomienda practicar un hemograma basal, el cual se debe repetir mensualmente durante el tratamiento y hasta 48 meses después de la última dosis. En caso de linfopenia grave, se debe proporcionar profilaxis para las infecciones herpéticas, reservando la suspensión del tratamiento para los casos de neutropenia o trombocitopenia grave ^71^.

En cuanto a la cladribina, también se observa linfopenia, al menos de grado 2, en el 90 % de los casos, llegando a ser de grado 3 en el 25,6 % y de grado 4 en el 0,7 % ^71^^,^^72^. Se sugiere la evaluación con un hemograma antes de cada dosis y hacer este control por laboratorio a los dos y a los seismeses de haber recibido el medicamento. Además, se debe confirmar que el número de linfocitos alcance a ser de, al menos, 800 por μl, antes de la administración de cada ciclo. Por último, se recomienda iniciar la profilaxis para las infecciones herpéticas si el número de linfocitos es inferior a 200 por μl ^71^^,^^72^.

Cabe aclarar que la mayoría de los datos previamente mencionados provienen de estudios pilares de investigación que tienen limitaciones, como muestras pequeñas y poblaciones no representativas que pueden no reflejar la diversidad de los pacientes de la práctica clínica cotidiana, especialmente en los países de ingresos de medianos a bajos, donde los recursos para la atención médica y el seguimiento pueden ser limitados ^44^^,^^81^. Esto enfatiza la necesidad de adelantar más investigaciones en condiciones del mundo real, para entender mejor la forma como la linfopenia y la suspensión de los tratamientos que modifican la enfermedad afectan diferentes subgrupos de pacientes.

En resumen, el recuento linfocitario proporciona información valiosa en los pacientes con esclerosis múltiple que reciben diferentes terapias modificadoras de la enfermedad. La linfopenia puede ser un efecto adverso frecuente con el dimetilfumarato y el fingolimod, y puede llevar a la suspensión del tratamiento. Sin embargo, en otros casos, como el de pacientes que reciben natalizumab y ocrelizumab, no es un efecto común, lo que requiere la búsqueda de otras causas. Es crucial reconocer y comprender estos fenómenos para llevar a cabo un seguimiento adecuado del número de linfocitos y adoptar las conductas apropiadas para cada paciente. Estas estrategias permiten la identificación temprana de los riesgos y la implementación de medidas preventivas adecuadas para minimizar las complicaciones infecciosas ^23^^,^^27^^,^^44^^,^^81^.

## ¿Cuál es la indicación de la terapia de células madre en la esclerosis múltiple?

El uso de tratamientos basados en células madre o células pluripotenciales para la esclerosis múltiple, ha avanzado significativamente desde sus inicios en la década de los 90. El trasplante de células madre hematopoyéticas es la modalidad con la cual se tiene la mayor experiencia clínica hasta el momento. Consiste en obtenerlas de sangre periférica y almacenarlas para su restitución mediante infusión después de usar un esquema terapéutico inmuno-mieloablativo, con el objetivo de reconstituir el sistema inmunitario y reducir su autorreactividad.

Se considera que el mecanismo de acción del trasplante de células madre hematopoyéticas en la esclerosis múltiple, consiste en la eliminación o reducción de las células inmunitarias patológicas, seguida de la reconstitución inmunitaria con células T y B restauradas ^82^. Este proceso favorece la regulación inmunitaria y disminuye la inflamación, eliminando las células inmunitarias autorreactivas y permitiendo la regeneración de un sistema inmunitario más tolerante ^83^.

Recientemente, las terapias basadas en células pluripotenciales mesenquimatosas ^83^ y en progenitores neurales buscan modular la respuesta inmunitaria y promover la reparación tisular, y han demostrado ser bien toleradas ^83^. Sin embargo, se requieren más estudios para determinar su eficacia clínica.

Dos estudios aleatorizados han demostrado la eficacia del trasplante de células madre hematopoyéticas en la esclerosis múltiple. En el estudio ASTIMS, un ensayo controlado aleatorizado multicéntrico de fase 2, se encontró que los pacientes tratados con este tipo de trasplante tenían menos lesiones hiperintensas en las secuencias T_2_ nuevas, en comparación con aquellos tratados con metotrexato ^84^. Además, en el seguimiento a cuatro años, ningún participante del grupo de trasplantes desarrolló lesiones activas, en contraste con el 56 % de los tratados con metotrexato (p = 0,029).

Clínicamente, la tasa anual de recaídas también fue significativamente menor en el grupo de trasplantes (0,19), en comparación con los del grupo de metotrexato (0,6) (OR = 0,36; IC_95%_: 0,15 a 0,88; p = 0,026). Sin embargo, en cuanto al cambio anual de la escala expandida del estado de discapacidad, no hubo una diferencia significativa ^84^.

Por su parte, en el estudio MIST, un ensayo controlado aleatorizado multicéntrico de fase 3, se demostró que el trasplante era más efectivo que los tratamientos que modifican la enfermedad (incluidos el natalizumab, el dimetilfumarato, el fingolimod, la mitoxantrona, la teriflunomida, el acetato de glatiramer y el interferón beta-1a) en los pacientes con esclerosis múltiple remitente recurrente ^85^. En el grupo de trasplantes, se encontró menor aumento de la discapacidad (5,8 % en el grupo de trasplantes frente a 69,2 % en los controles [OR = 0,07; IC_95%_: 0,02 a 0,24; p < 0,001]), así como menor tasa de recaídas y un tiempo más prolongado hasta la progresión de la enfermedad ^85^. Múltiples estudios observacionales han añadido evidencia a favor de su efectividad, en comparación con las diferentes terapias modificadoras de la enfermedad ^84^^-^^86^.

Es esencial ser críticos de los estudios que respaldan estos hallazgos. Si bien los estudios aleatorizados aportan información valiosa, la falta de diversidad en el muestreo de pacientes puede limitar la generalización de sus resultados. Las discrepancias en las medidas de eficacia, como el cambio en la escala expandida del estado de discapacidad, sugieren que, si bien el trasplante puede ser superior en algunos aspectos, no aborda todas las dimensiones del manejo de la esclerosis múltiple. Además, muchos estudios observacionales, aunque informativos, pueden sufrir sesgos de selección y es posible que se no controlen adecuadamente las variables de confusión ^87^.

Estos puntos resaltan la necesidad de más investigaciones que validen y amplíen estos resultados en los contextos locales y en los países de ingresos medios y bajos, donde el acceso a los tratamientos avanzados, a menudo, es limitado. En Latinoamérica, la frecuencia de trasplantes es entre 20 y 40 veces menor en comparación con Europa y Norteamérica. Factores como la organización del sistema de salud, la disponibilidad de infraestructura y el acceso a los recursos necesarios para realizar trasplantes de células madre, así como la capacitación del personal médico, son factores determinantes fundamentales para la implementación exitosa de este tratamiento ^88^.

En conclusión, el trasplante de células madre hematopoyéticas es una opción razonable para los pacientes con esclerosis múltiple remitente recurrente muy activa, que no responden a otras terapias modificadoras de la enfermedad de gran eficacia. También, podría considerarse como una opción para los pacientes con esclerosis múltiple agresiva sin tratamiento previo, o en el contexto de estudios clínicos ^86^. En términos de seguridad, la selección de los pacientes es fundamental para aumentar la probabilidad de beneficio y reducir el riesgo de efectos adversos. Las características de los pacientes con mayores probabilidades de beneficiarse del trasplante incluyen: edad menor de 50 años, inicio relativamente reciente de la enfermedad (duración menor de 15 años), discapacidad leve a moderada, cursos de la enfermedad muy activos y persistencia de actividad de la enfermedad a pesar de recibir otros tratamientos de gran eficacia que modifican la enfermedad ^86^.

## ¿Cómo evitar el efecto de rebote al suspender algunas terapias modificadoras de la enfermedad?

La elección del mejor de estos tratamientos para cada paciente con esclerosis múltiple es uno de los principales retos para el médico tratante tras el establecimiento del diagnóstico. Se ha documentado que, aproximadamente, el 50 % de los pacientes requerirá un cambio de la primera molécula administrada después de tres años de tratamiento, debido a diversos factores que incluyen intolerancia, falta de eficacia, deseo de concebir, dificultad para la dispensación y problemas para cumplir con las dosis y administración del medicamento ^89^.

Cuando se cambia un tratamiento, un periodo de lavado (o periodo de descanso farmacológico) corto se ha relacionado con un mayor riesgo de reacciones adversas, principalmente infecciosas. Por el contrario, un periodo prolongado de lavado se asocia con un mayor riesgo de efecto de rebote. Este efecto se define como una actividad clínica o imagenológica de la enfermedad, que se considera desproporcionada en comparación con el patrón observado antes del inicio del medicamento suspendido ^90^. Según los estudios realizados, principalmente en cohortes caucásicas, entre las terapias modificadoras de la enfermedad, el natalizumab y el fingolimod son los fármacos que con mayor frecuencia se asocian con este fenómeno.

Para el fingolimod, se ha calculado un aumento en la tasa anual de recaídas del 0,37 al 0,47 tras la suspensión del medicamento y la mayoría de los eventos ocurre en los primeros tres meses ^90^. En las poblaciones evaluadas, se ha observado que los pacientes afectados con mayor frecuencia son aquellos que son más jóvenes en el momento del diagnóstico de la enfermedad, los que han estado expuestos al fingolimod durante más tiempo y quienes han presentado recuentos linfocitarios más bajos ^91^.

En cuanto a la suspensión del natalizumab, se ha observado una probabilidad acumulada del fenómeno de rebote del 39 %, la mayoría de los eventos ocurre entre los tres y los nueve meses después de su suspensión y existe una asociación con recaídas graves en el 9 % de los pacientes ^92^^,^^93^. En un estudio en el que se incluyeron 14 213 pacientes con 18 029 episodios de suspensión de diferentes terapias modificadoras de la enfermedad, se identificaron los siguientes factores predictores del fenómeno de rebote: alta tasa de recaídas el año anterior a la suspensión del medicamento, sexo femenino, pacientes más jóvenes y puntajes elevados en la escala expandida del estado de discapacidad (evidencia de clase III) ^19^.

Se han planteado varias estrategias sobre cuándo y cómo suspender de manera segura el fingolimod y el natalizumab, para evitar el efecto de rebote. En general, se ha propuesto cambiar el tratamiento modificador de la enfermedad de manera vertical en función de una mayor actividad de la enfermedad, de forma rápida y directa a un fármaco de mayor eficacia, o de manera horizontal considerando alternativas de eficacia similar y, en algunos casos, con una transición más gradual, si la razón para suspender las moléculas no está relacionada con la necesidad de un medicamento más eficaz ^94^.

Además, se recomienda no usar un periodo de lavado al suspender el fingolimod o el natalizumab para cambiar a interferón ß1a, acetato de glatiramer, teriflunomida o dimetilfumarato. Si se desea comenzar la administración de ocrelizumab, cladribina o alemtuzumab, se sugiere un periodo de lavado de entre uno y tres meses ^94^.

Para todos los cambios de medicamentos, se deben mantener los periodos de lavado lo más cortos posibles, individualizando los factores de riesgo y las particularidades de cada paciente, e identificando de manera temprana a aquellos con mayores probabilidades de presentar el efecto de rebote. El uso de esteroides durante el proceso de transición entre los tratamientos que modifican la enfermedad para la esclerosis múltiple ha sido presentado de manera anecdótica por algunos centros y no se recomienda de forma rutinaria ^95^.

En el contexto colombiano y de otros países donde se presentan dificultades para la dispensación periódica de este tipo de tratamientos, podrían presentarse con mayor frecuencia situaciones en las que los pacientes experimentan fenómenos de rebote. Por esta razón, en nuestro medio se deben ampliar las consideraciones de los riesgos frente a los beneficios al prescribir los tratamientos, en especial, cuando se trata de moléculas con mayor riesgo de presentar el fenómeno de rebote después de su suspensión. Por ejemplo, en los pacientes latinoamericanos, el fingolimod ha mostrado tener un perfil de seguridad y eficacia similar al de otras poblaciones ^96^, mientras que el natalizumab se asocia con menores costos y un mayor impacto en los años de vida ajustados por calidad (AVAC) en los pacientes con esclerosis múltiple remitente recurrente muy activa ^97^.

En conclusión, las estrategias para la suspensión y el cambio de tratamientos son cruciales, ya que el prolongar el periodo de lavado innecesariamente puede resultar en un aumento del riesgo de reactivación de la enfermedad. En la literatura actual, se evidencia que, entre las terapias modificadoras de la enfermedad disponibles, el fingolimod y el natalizumab son las moléculas con un mayor riesgo de rebote, lo que pone de manifiesto la importancia de cambiar estos tratamientos de manera planificada, favoreciendo un enfoque individualizado que considere las características y necesidades particulares de cada paciente.

## References

[1] Coyle PK (2021). What can we learn from sex differences in MS?. J Pers Med.

[2] Reyes MA, Vicuña J, Navas Á (2016). Esclerosis múltiple y embarazo. Revista Repertorio de Medicina y Cirugía.

[3] Toro J, Cárdenas S, Martínez F, Urrutia J, Díaz C (2013). Multiple sclerosis in Colombia and other Latin American Countries. Mult Scler Relat Disord.

[4] Ordóñez G, Romero S, Orozco L, Pineda B, Jiménez-Morales S, Nieto A (2015). Genomewide admixture study in Mexican mestizos with multiple sclerosis. Clin Neurol Neurosurg.

[5] Confavreux C, Hutchinson M, Hours MM, Cortinovis-Tourniaire P, Moreau T (1998). Rate of pregnancy-related relapse in multiple sclerosis. Pregnancy in Multiple Sclerosis Group. N Engl J Med.

[6] De Seze J, Chapelotte M, Delalande S, Ferriby D, Stojkovic T, Vermersch P (2004). Intravenous corticosteroids in the postpartum period for reduction of acute exacerbations in multiple sclerosis. Mult Scler J.

[7] Ávila-Ornelas J, Ávila M, Stosic M, Robles L, Prieto PG, Hutton GJ (2011). The role of postpartum intravenous corticosteroids in the prevention of relapses in multiple sclerosis. Int J MS Care.

[8] Leguy S, Lefort M, Lescot L, Michaud A, Vukusic S, Le Page E (2022). COPP-MS: Corticosteroids during the post-partum in relapsing multiple sclerosis patients. J Neurol.

[9] Finkelsztejn A, Fragoso YD, Ferreira MLB, Lana-Peixoto MA, Alves-Leon SV, Gomes S (2011). The Brazilian database on pregnancy in multiple sclerosis. Clin Neurol Neurosurg.

[10] Rosa GR, O’Brien AT, Nogueira EDAG, Carvalho VMD, Paz SC, Fragoso YD (2018). There is no benefit in the use of postnatal intravenous immunoglobulin for the prevention of relapses of multiple sclerosis: findings from a systematic review and meta-analysis. Arq Neuropsiquiatr.

[11] Menascu S, Siegel-Kirshenbaum M, Dreyer-Alster S, Warszawer Y, Magalashvili D, Dolev M (2023). Intravenous immunoglobulin treatment during pregnancy and the post-partum period in women with multiple sclerosis: A prospective analysis. Mult Scler J Exp Transl Clin.

[12] Vukusic S, Ionescu I, Cornu C, Bossard N, Durand-Dubief F, Cotton F (2021). Oral nomegestrol acetate and transdermal 17-beta-estradiol for preventing post-partum relapses in multiple sclerosis: The POPARTMUS study. Mult Scler J.

[13] Krysko KM, Rutatangwa A, Graves J, Lazar A, Waubant E (2020). Association between breastfeeding and postpartum multiple sclerosis relapses: a systematic review and metaanalysis. JAMA Neurol.

[14] Schubert C, Steinberg L, Peper J, Ramien C, Hellwig K, Köpke S (2023). Postpartum relapse risk in multiple sclerosis: a systematic review and meta-analysis. J Neurol Neurosurg Psychiatry.

[15] Haben S, Ciplea AI, Tokic M, Timmesfeld N, Thiel S, Gold R (2024). Early postpartum treatment strategies and early postpartum relapses in women with active multiple sclerosis. J Neurol Neurosurg Psychiatry.

[16] Yeh WZ, Widyastuti PA, Van Der Walt A, Stankovich J, Havrdova E, Horakova D (2021). Natalizumab, fingolimod, and dimethyl fumarate use and pregnancy-related relapse and disability in women with multiple sclerosis. Neurology.

[17] Galati A, McElrath T, Bove R (2022). Use of B-cell-depleting therapy in women of childbearing potential with multiple sclerosis and neuromyelitis optica spectrum disorder. Neurol Clin Pract.

[18] Bove R, Applebee A, Bawden K, Fine C, Shah A, Avila RL (2024). Patterns of diseasemodifying therapy utilization before, during, and after pregnancy and postpartum relapses in women with multiple sclerosis. Mult Scler Relat Disord.

[19] Roos I, Malpas C, Leray E, Casey R, Horakova D, Havrdova EK (2022). Disease reactivation after cessation of disease-modifying therapy in patients with relapsing-remitting multiple sclerosis. Neurology.

[20] Hellwig K, Tokic M, Thiel S, Hemat S, Timmesfeld N, Ciplea AI (2023). Multiple sclerosis disease activity and disability following cessation of fingolimod for pregnancy. Neurol Neuroimmunol Neuroinflammation.

[21] Gklinos P, Dobson R (2023). Monoclonal antibodies in pregnancy and breastfeeding in patients with multiple sclerosis: A review and an updated clinical guide. Pharmaceuticals.

[22] Graham EL, Bove R, Costello K, Crayton H, Jacobs DA, Shah S (2024). Practical considerations for managing pregnancy in patients with multiple sclerosis: Dispelling the myths. Neurol Clin Pract.

[23] Dobson R, Dassan P, Roberts M, Giovannoni G, Nelson-Piercy C, Brex PA (2019). UK consensus on pregnancy in multiple sclerosis: Association of British Neurologists guidelines. Pract Neurol.

[24] Vukusic S, Carra-Dalliere C, Ciron J, Maillart E, Michel L, Leray E (2023). Pregnancy and multiple sclerosis: 2022 recommendations from the French multiple sclerosis society. Mult Scler.

[25] Vukusic S, Marignier R, Ciron J, Bourre B, Cohen M, Deschamps R (2022). Pregnancy and neuromyelitis optica spectrum disorders: 2022 recommendations from the French Multiple Sclerosis Society. Mult Scler.

[26] Otero-Romero S, Lebrun-Frénay C, Reyes S, Amato MP, Campins M, Farez M (2023). European Committee for Treatment and Research in Multiple Sclerosis and European Academy of Neurology consensus on vaccination in people with multiple sclerosis: Improving immunization strategies in the era of highly active immunotherapeutic drugs. Eur J Neurol.

[27] Reyes S, Ramsay M, Ladhani S, Amirthalingam G, Singh N, Cores C (2020). Protecting people with multiple sclerosis through vaccination. Pract Neurol.

[28] Nour MM, Nakashima I, Coutinho E, Woodhall M, Sousa F, Revis J (2016). Pregnancy outcomes in aquaporin-4-positive neuromyelitis optica spectrum disorder. Neurology.

[29] Davoudi V, Keyhanian K, Bove RM, Chitnis T (2016). Immunology of neuromyelitis optica during pregnancy. Neurol Neuroimmunol Neuroinflamm.

[30] Budtarad N, Ongphichetmehta T, Siritho S (2025). Insights into neuromyelitis optica spectrum disorder and pregnancy from a single-center study in Thailand. Sci Rep.

[31] Varytė G, Arlauskienė A, Ramašauskaitė D (2021). Pregnancy and multiple sclerosis: An update. Curr Opin Obstet Gynecol.

[32] Lavie C, Rollot F, Durand-Dubief F, Marignier R, Ionescu I, Casey R (2019). Neuraxial analgesia is not associated with an increased risk of post-partum relapses in MS. Mult Scler.

[33] Vukusic S, Bove R, Dobson R, McElrath T, Oreja-Guevara C, Pietrasanta C (2025). Pregnancy and infant outcomes in women with multiple sclerosis treated with ocrelizumab. Neurol Neuroimmunol Neuroinflammation.

[34] Giovannoni G, Galazka A, Schick R, Leist T, Comi G, Montalban X (2020). Pregnancy outcomes during the clinical development program of cladribine in multiple sclerosis: An integrated analysis of safety. Drug Saf.

[35] Capone F, Albanese A, Quadri G, Di Lazzaro V, Falato E, Cortese A (2022). Diseasemodifying drugs and breastfeeding in multiple sclerosis: A narrative literature review. Front Neurol.

[36] Dost-Kovalsky K, Thiel S, Ciplea AI, Gold R, Hellwig K (2023). Cladribine and pregnancy in women with multiple sclerosis: The first cohort study. Mult Scler.

[37] Jacobs BM, Peter M, Giovannoni G, Noyce AJ, Morris HR, Dobson R (2022). Towards a global view of multiple sclerosis genetics. Nat Rev Neurol.

[38] Balcerac A, Louapre C (2022). Genetics and familial distribution of multiple sclerosis: A review. Rev Neurol.

[39] Kim W, Patsopoulos NA (2022). Genetics and functional genomics of multiple sclerosis. Semin Immunopathol.

[40] Toro J, Cuéllar-Giraldo D, Díaz-Cruz C, Burbano LE, Guío CM, Reyes S (2016). HLADRB1* 14 is a protective allele for multiple sclerosis in an admixed Colombian population. Neurol Neuroimmunol Neuroinflamm.

[41] Goris A, Vandebergh M, McCauley JL, Saarela J, Cotsapas C (2022). Genetics of multiple sclerosis: Lessons from polygenicity. Lancet Neurol.

[42] International Multiple Sclerosis Genetics Consortium, MultipleMS Consortium (2023). Locus for severity implicates CNS resilience in progression of multiple sclerosis. Nature.

[43] Barrie W, Yang Y, Irving-Pease EK, Attfield KE, Scorrano G, Jensen LT (2024). Elevated genetic risk for multiple sclerosis emerged in steppe pastoralist populations. Nature.

[44] Rivas V, Flores JDJ, Rito Y, Corona T (2017). The genetics of multiple sclerosis in Latin America. Mult Scler.

[45] Rivera VM (2017). Multiple sclerosis in Latin Americans: Genetic aspects. Curr Neurol Neurosci Rep.

[46] Paradela ER, Alves-Leon SV, Figueiredo ALS, Pereira VCSR, Malfetano F, Mansur LF (2015). The CIITA genetic polymorphism rs4774*C in combination with the HLA-DRB1*15:01 allele as a putative susceptibility factor to multiple sclerosis in Brazilian females. Arq Neuropsiquiatr.

[47] Patrucco L, Larriba J, Redal MA, Rojas JI, Argibay PF, Cristiano E (2009). HLA-DRB1 and multiple sclerosis in Argentina. Eur J Neurol.

[48] Jokubaitis VG, Campagna MP, Ibrahim O, Stankovich J, Kleinova P, Matesanz F (2023). Not all roads lead to the immune system: The genetic basis of multiple sclerosis severity. Brain.

[49] Loginovic P, Wang F, Li J, Ferrat L, Mirshahi UL, Rao HS (2024). Applying a genetic risk score model to enhance prediction of future multiple sclerosis diagnosis at first presentation with optic neuritis. Nat Commun.

[50] Ferreira-Atuesta C, Reyes S, Giovanonni G, Gnanapavan S (2021). The evolution of neurofilament light chain in multiple sclerosis. Front Neurosci.

[51] Melamed E, Lee MW (2020). Multiple sclerosis and cancer: The ying-yang effect of disease modifying therapies. Front Immunol.

[52] Kingwell E, Evans C, Zhu F, Oger J, Hashimoto S, Tremlett H (2014). Assessment of cáncer risk with β-interferon treatment for multiple sclerosis. J Neurol Neurosurg Psychiatry.

[53] Dolladille C, Chrétien B, Peyro-Saint-Paul L, Alexandre J, Dejardin O, Fedrizzi S (2021). Association between disease-modifying therapies prescribed to persons with multiple sclerosis and cancer: A who pharmacovigilance database analysis. Neurotherapeutics.

[54] Lebrun C, Rocher F (2018). Cancer risk in patients with multiple sclerosis: Potential impact of disease-modifying drugs. CNS Drugs.

[55] Vermersch P, Czlonkowska A, Grimaldi LM, Confavreux C, Comi G, Kappos L (2014). Teriflunomide versus subcutaneous interferon beta-1a in patients with relapsing multiple sclerosis: A randomised, controlled phase 3 trial. Mult Scler J.

[56] O’Connor P, Wolinsky JS, Confavreux C, Comi G, Kappos L, Olsson TP (2011). Randomized trial of oral teriflunomide for relapsing multiple sclerosis. N Engl J Med.

[57] Gold R, Kappos L, Arnold DL, Bar-Or A, Giovannoni G, Selmaj K (2012). Placebo-controlled phase 3 study of oral bg-12 for relapsing multiple sclerosis. N Engl J Med.

[58] Calabresi PA, Radue EW, Goodin D, Jeffery D, Rammohan KW, Reder AT (2014). Safety and efficacy of fingolimod in patients with relapsing-remitting multiple sclerosis (FREEDOMS II): A double-blind, randomised, placebo-controlled, phase 3 trial. Lancet Neurol.

[59] Lublin F, Miller DH, Freedman MS, Cree BAC, Wolinsky JS, Weiner H (2016). Oral fingolimod in primary progressive multiple sclerosis (INFORMS): A phase 3, randomised, double-blind, placebo-controlled trial. Lancet.

[60] Pakpoor J, Disanto G, Altmann DR, Pavitt S, Turner BP, Marta M (2015). No evidence for higher risk of cancer in patients with multiple sclerosis taking cladribine. Neurol Neuroimmunol Neuroinflamm.

[61] Polman CH, O’Connor PW, Havrdova E, Hutchinson M, Kappos L, Miller DH (2006). A randomized, placebo-controlled trial of natalizumab for relapsing multiple sclerosis.. N Engl J Med.

[62] Cohen JA, Coles AJ, Arnold DL, Confavreux C, Fox EJ, Hartung HP (2012). Alemtuzumab versus interferon beta 1a as first-line treatment for patients with relapsing-remitting multiple sclerosis: A randomised controlled phase 3 trial. Lancet.

[63] Havrdova E, Arnold DL, Cohen JA, Hartung HP, Fox EJ, Giovannoni G (2018). Alemtuzumab CARE-MS I 5-year follow-up: Durable efficacy in the absence of continuous MS therapy. Neurology.

[64] Hauser SL, Bar-Or A, Comi G, Giovannoni G, Hartung HP, Hemmer B (2017). Ocrelizumab versus interferon beta-1a in relapsing multiple sclerosis. N Engl J Med.

[65] Montalbán X, Hauser SL, Kappos L, Arnold DL, Bar-Or A, Comi G (2017). Ocrelizumab versus placebo in primary progressive multiple sclerosis. N Engl J Med.

[66] Hauser SL, Zielman R, Das Gupta A, Xi J, Stoneman D, Karlsson G (2023). Efficacy and safety of four-year ofatumumab treatment in relapsing multiple sclerosis: The ALITHIOS open-label extension. Mult Scler J.

[67] Kister I (2017). Disease-modifying therapies can be safely discontinued in an individual with stable relapsing-remitting MS - YES. Mult Scler J.

[68] Corboy JR, Fox RJ, Kister I, Cutter GR, Morgan CJ, Seale R (2023). Risk of new disease activity in patients with multiple sclerosis who continue or discontinue disease-modifying therapies (DISCOMS): A multicentre, randomised, single-blind, phase 4, non-inferiority trial. Lancet Neurol.

[69] Prosperini L, Haggiag S, Ruggieri S, Tortorella C, Gasperini C (2023). Stopping disease-modifying treatments in multiple sclerosis: A systematic review and meta-analysis of real-world studies. CNS Drugs.

[70] Fox EJ, Buckle GJ, Singer B, Singh V, Boster A (2019). Lymphopenia and DMTs for relapsing forms of MS: Considerations for the treating neurologist. Neurol Clin Pract.

[71] Fischer S, Proschmann U, Akgün K, Ziemssen T (2021). Lymphocyte counts and multiple sclerosis therapeutics: between mechanisms of action and treatment-limiting side effects. Cells.

[72] Schweitzer F, Laurent S, Fink GR, Barnett MH, Hartung HP, Warnke C. (2021). Effects of diseasemodifying therapy on peripheral leukocytes in patients with multiple sclerosis. J Neurol.

[73] Anand P (2021). Neurologic infections in patients on immunomodulatory and immunosuppressive therapies. Continuum.

[74] Barboza A, Sinay V, Alonso R, Carnero-Contentti E, Hryb J, Silva B (2024). Comorbidities in multiple sclerosis and their influence on the choice of treatment. Rev Neurol.

[75] Beishuizen SJE, Geerlings SE (2009). Immune reconstitution inflammatory syndrome: immunopathogenesis, risk factors, diagnosis, treatment and prevention. Neth J Med.

[76] McGuire JL, Fridman V, Wüthrich C, Koralnik IJ, Jacobs D (2011). Progressive multifocal leukoencephalopathy associated with isolated CD8+ T-lymphocyte deficiency mimicking tumefactive MS. J Neurovirol.

[77] Elgenidy A, Abdelhalim NN, Al-Kurdi MAM, Mohamed LA, Ghoneim MM, Fathy AW (2024). Hypogammaglobulinemia and infections in patients with multiple sclerosis treated with anti-CD20 treatments: A systematic review and meta-analysis of 19,139 multiple sclerosis patients. Front Neurol.

[78] Álvarez E, Longbrake EE, Rammohan KW, Stankiewicz J, Hersh CM (2023). Secondary hypogammaglobulinemia in patients with multiple sclerosis on anti-CD20 therapy: Pathogenesis, risk of infection, and disease management. MSRD.

[79] Talah BA, Mello J, De Oliveira Carriço HRM, Argota NT, Tanimoto L, Teles M (2025). Risk of hypogammaglobulinemia and infections associated with rituximab therapy in patients with multiple sclerosis: A systematic review and meta-analysis (P8-1.013). Neurology.

[80] Gomes ABA, Bueno L, Silva GD, Diniz CC, Barbosa R, Lara AN (2022). Reducing infection risk in multiple sclerosis and neuromyelitis optica spectrum disorders: A Brazilian reference center’s approach. Arq Neuropsiquiatr.

[81] Calocer F, Dejardin O, Droulon K, Launoy G, Defer G (2018). Socio-economic status influences access to second-line disease modifying treatment in Relapsing Remitting Multiple Sclerosis patients. PLoS One.

[82] Petrou P, Kassis I, Levin N, Paul F, Backner Y, Benoliel T (2020). Beneficial effects of autologous mesenchymal stem cell transplantation in active progressive multiple sclerosis. Brain.

[83] Genchi A, Brambilla E, Sangalli F, Radaelli M, Bacigaluppi M, Furlan R (2023). Neural stem cell transplantation in patients with progressive multiple sclerosis: An open-label, phase 1 study. Nat Med.

[84] Mancardi G, Sormani M, Di Gioia M, Vuolo L, Gualandi F, Amato M (2012). Autologous haematopoietic stem cell transplantation with an intermediate intensity conditioning régimen in multiple sclerosis: The Italian multi-centre experience. Mult Scler.

[85] Burt RK, Balabanov R, Burman J, Sharrack B, Snowden JA, Oliveira MC (2019). Effect of nonmyeloablative hematopoietic stem cell transplantation vs continued disease-modifying therapy on disease progression in patients with relapsing-remitting multiple sclerosis: a randomized clinical trial. JAMA.

[86] Ross LA, Stropp LM, Cohen JA (2024). Autologous hematopoietic stem cell transplantation to treat multiple sclerosis. Neurol Clin.

[87] Flores-Alfaro E, Burguete-García AI, Salazar-Martínez E (2012). Diseños de investigación en epidemiología genética. Rev Panam Salud Públ.

[88] Gale RP, Seber A, Bonfim C, Pasquini M (2016). Haematopoietic cell transplants in Latin America. Bone Marrow Transplant.

[89] Signoriello E, Landi D, Monteleone F, Saccà F, Nicoletti CG, Buttari F (2018). Fingolimod reduces the clinical expression of active demyelinating lesions in MS. MSRD.

[90] Barry B, Erwin AA, Stevens J, Tornatore C (2019). Fingolimod rebound: A review of the clinical experience and management considerations. Neurol Ther.

[91] Maunula A, Atula S, Laakso S, Tienari P (2024). Frequency and risk factors of rebound after fingolimod discontinuation - A retrospective study. MSRD.

[92] Gueguen A, Roux P, Deschamps R, Moulignier A, Bensa C, Savatovsky J (2014). Abnormal inflammatory activity returns after natalizumab cessation in multiple sclerosis. JNNP.

[93] Fagius J, Feresiadou A (2017). Larsson esclerosis múltiple, Burman J. Discontinuation of disease modifying treatments in middle aged multiple sclerosis patients. First line drugs vs natalizumab. MSRD.

[94] Bigaut K, Cohen M, Durand-Dubief F, Maillart E, Planque E, Zephir H (2021). How to switch disease-modifying treatments in multiple sclerosis: Guidelines from the French Multiple Sclerosis Society (SFSEP). MSRD.

[95] Borriello G, Prosperini L, Mancinelli C, Giannì C, Fubelli F, Pozzilli C. (2012). Pulse monthly steroids during an elective interruption of natalizumab: a post-marketing study. Eur J Neurol.

[96] Ordóñez-Boschetti L, Rey R, Cruz A, Sinha A, Reynolds T, Frider N (2015). Safety and tolerability of fingolimod in Latin American patients with relapsing-remitting multiple sclerosis: The Open-Label FIRST LATAM Study. Adv Ther.

[97] Lasalvia P, Hernández F, Castañeda-Cardona C, Cuestas JA, Rosselli D (2020). Cost-effectiveness of natalizumab compared with fingolimod for relapsing-remitting multiple sclerosis treatment in Colombia. ViHRI.

